# Complete genome sequencing and assessment of mutation-associated protein dynamics of the first Indian bovine ephemeral fever virus (BEFV) isolate

**DOI:** 10.1080/01652176.2021.1995909

**Published:** 2021-10-29

**Authors:** Shruti Pyasi, Advika Gupta, Nagendra R. Hegde, Debasis Nayak

**Affiliations:** a Discipline of Bioscience and Biomedical Engineering, Indian Institute of Technology Indore, Indore, India; b Department of Biotechnology, National Institute of Animal Biotechnology, Hyderabad, India

**Keywords:** Bovine, cattle, bovine ephemeral fever virus, complete genome, BEFV, Ephemerovirus, mutations, protein dynamics, phylogeny, India

## Abstract

**Background:**

Bovine ephemeral fever (BEF) is a re-emerging disease caused by bovine ephemeral fever virus (BEFV). Although it poses a huge economic threat to the livestock sector, complete viral genome information from any South Asian country, including India, lacks.

**Aim:**

Genome characterization of the first Indian BEFV isolate and to evaluate its genetic diversity by characterizing genomic mutations and their associated protein dynamics.

**Materials and Methods:**

Of the nineteen positive blood samples collected from BEF symptomatic animals during the 2018-19 outbreaks in India, one random sample was used to amplify the entire viral genome by RT-PCR. Utilizing Sanger sequencing and NGS technology, a complete genome was determined. Genome characterization, genetic diversity and phylogenetic analyses were explored by comparing the results with available global isolates. Additionally, unique genomic mutations within the Indian isolate were investigated, followed by *in-silico* assessment of non-synonymous (NS) mutations impacts on corresponding proteins’ secondary structure, solvent accessibility and dynamics.

**Results:**

The complete genome of Indian BEFV has 14,903 nucleotides with 33% GC with considerable genetic diversity. Its sequence comparison and phylogenetic analysis revealed a close relatedness to the Middle Eastern lineage. Genome-wide scanning elucidated 30 unique mutations, including 10 NS mutations in the *P, L* and *G_NS_* proteins. The mutational impact evaluation confirmed alterations in protein structure and dynamics, with minimal effect on solvent accessibility. Additionally, alteration in the interatomic interactions was compared against the wild type.

**Conclusion:**

These findings extend our understanding of the BEFV epidemiological and pathogenic potential, aiding in developing better therapeutic and preventive interventions.

## 1. Introduction

Bovine ephemeral fever virus (BEFV) is a widespread and economically significant bovine pathogen associated with bovine ephemeral fever (BEF) disease. The first report on the emergence of BEFV outbreaks dates back to the mid-nineteenth century in Africa, which became endemic to Australia, Africa, the Middle East, and major parts of the Asiatic continent, including East Asia and South-East Asia (Niwa et al. 2015). The outbreaks persist during monsoon onset, coinciding with vectors propagation (culicoid midges and mosquitoes) that spreads the disease (Venter et al. 2003). The clinical manifestations of BEF include polyphasic fever, synovitis, somnolence, muscle stiffness, shifting lameness, reluctance to move, recumbency, and inappetence (St. George b^1986^). Presently enzootic, the disease is characterized by high morbidity and occasional high case fatality (2-20%). The disease causes enormous economic loss arising from a reduction in milk production, impaired reproduction, traction decline, loss of body weight, and livestock-associated trade restrictions (Hsieh et al. 2005; Tonbak et al. 2013).

The BEFV belongs to the genus *Ephemerovirus* of the *Rhabdoviridae* family. It is an enveloped, bullet-shaped virion that enwraps an ∼14.9 kb, non-segmented (-) RNA genome encoding ten transcription units in the order 3′-*l*-*N-P-M-G*-[*G_NS_*-*α1-α2-β-γ*]-*L*-*t*-5′ (McWilliam et al. 1997). The virion contains five structural proteins, namely, nucleoprotein (*N*, 52kD), phosphoprotein (*P*, 43 kDa), matrix protein (*M*, 29 kDa), glycoprotein (*G*, 81 kDa), large polymerase protein (*L,* 180 kDa). Additionally, genome codes for a non-structural glycoprotein (G*_NS_*, 90 kDa) and few accessory proteins *α, β* and *γ* (each of <15kDa) (Walker and Klement 2015). Each gene junctions inhabit intergenic regions (IGRs), flanked by regulatory motifs termed transcription initiation (TI), transcription termination/polyadenylation (TTP) signals (Rose 1980; Barr et al. 1997). This arrangement likely regulates the sequential transcription by a “stop-start” process resulting in a 3′→5′ polar gradient of mRNA synthesis (Dietzgen et al. 2017). The phylogenetic analyses based on the glycoprotein (*G*) gene delineates circulating BEFV into geographically distinct Australian, Middle Eastern, Southeast Asian, and newly emerging South African lineages (Walker and Klement 2015; Omar et al. 2020). Although BEFV is serologically monotypic (Walker and Klement 2015), its diverse distribution and differential pathogenicity indicate its genetic variability. The mutation accumulation probability among all RNA viruses is driven by multiple factors, such as poor proofreading by polymerase protein, replicative repairing, selective pressure, etc. (Sardar et al. 2020; Wang et al. 2020). Notably, mutations could lead to protein structural alterations that affect the viral immunological properties, transmission, and pathogenicity (Woźniakowski et al. 2009; Sardar et al. 2020). Previous studies have suggested BEFV to possess high substitution rates (Trinidad et al. 2014). However, few recent reports on the generation of novel genogroups with higher viral virulence (Yanase et al. 2020) and limited vaccine effectiveness suggest BEFV evolution is enhancing variant's fitness landscape to help overcome the host immune system (Aziz-Boaron et al. 2014).

The new geographical expansions with enhanced case fatality rate and high production losses alarm BEFV as an evolving arbovirus that warrants closer scrutiny in surveillance, epidemiology, and ecological impact (Lee 2019; Yanase et al. 2020). Despite the multiple decades of episodic reporting on seasonal incursions from many South Asian countries, including India (Nandi and Negi 1999; Walker and Klement 2015), no complete sequence information was available until recently (Pyasi et al. 2020), which hinders progress for its detection and prevention. Field detection solely relies on the observed clinical signs accompanied by haemato-biochemical analysis in the lack of any serological diagnostics kits. However, molecular diagnosis affirms the viral suspicion. Thus, the current study aimed at genome sequencing and characterizing the first complete Indian BEFV genome. It further analysed its genetic diversity by investigating genomic mutations and their associated protein dynamics that could provide better insight into the viral pathobiology.

## 2. Materials and methods

### 2.1. Field sample collection

Blood samples (n = 25) were collected from symptomatic animals during the 2018–19 outbreaks in Madhya Pradesh, India. Bovine aged between 8 months to 8 years of age showed prominent clinical signs in the study. Veterinary professionals collected the blood sample. Haematological and biochemical analysis was performed with/without anticoagulants as required. Subsequently, serum and peripheral blood mononuclear cells (PBMCs) were separated by centrifugation at 3,000 rpm for 15 minutes and kept at −80 °C until future use.

### 2.2. RNA extraction and genome sequencing

Viral RNA was extracted from serum utilising TRIzol method (Invitrogen, Waltham, MA, USA) and converted to cDNA using Prime Script cDNA synthesis kit (Takara Bio Inc, Kusatsu, Japan). The viral presence was confirmed by reverse transcriptase PCR (RT-PCR) using published primers (Zheng and Qiu 2012) with previously described PCR conditions (Hsieh et al. 2005). Subsequently, overlapping RT-PCR using eleven primer pairs, designed as per the conserved regions of reference strain (accession no: NC_002526), was performed with optimized annealing between 55 °C and 62 °C as per the primer set (Table S1). Purified amplicons were directly sequenced using the ABI3100 platform (Applied Biosystems, USA). All sequences were subjected to Blastn search and assembled using BioEdit (Tom et al. 2011).

Alternatively, sequence accuracy was determined by performing Illumina sequencing, a commonly used next-generation sequencing (NGS) technology. Libraries preparation was done using total viral RNA with NEBNext mRNA Library Prep kit (NEB, Ipswich, MA, USA) and sequenced in a MiSeq instrument (150 cycles, paired-end sequencing). The sequenced raw data followed trimming and adapter removal using Trim Galore (Krueger 2015). Further, it reads classification by Kraken-2 utilizing a standard database (Wood et al. 2019). The only confirmed viral reads were snipped against other non-viral reads by BBMap (Bushnell 2014) and assembled into contigs using MEGAHIT v.1.1.3 (Li et al. 2015) to generate a *de novo* sequence.

### 2.3. Phylogenetic and genetic analysis

Phylogenetic analyses were inferred by the maximum likelihood method employing the Tamura-Nei model of nt substitution model using the MEGA X (1000 bootstrap replicates) (Kumar et al. 2018). Furthermore, the new genome sequence was aligned with all available BEFV complete genomes archived from GenBank using the ClustalW (Larkin et al. 2007) module of BioEdit to determine terminal sequence conservancy, molecular characterization, and relative similarity evaluation. Eventually, genetic variations among the consensus sequences in addition to the novel nucleotide (nt) and amino acid (aa) substitutions were inspected as synonymous (SN) and non-synonymous (NS). Lastly, the effect of NS mutations on structural alteration was evaluated.

### 2.4. Mutational assessment

The detected genomic mutations, emphasizing unique NS mutations, were examined to better understand the mutational consequences on protein structure and dynamics. *P, L* and *G_NS_* proteins of Indian BEFV isolate that possessed such mutations were extensively investigated utilizing several computational approach-based tools. We considered the first globally reported Australian isolate as wild type sequence (AF234533) for all comparisons.

#### 2.4.1. Secondary structures prediction

The secondary structure of investigated proteins was predicted by CFSSP: Chou and Fasman secondary structure prediction server (Kumar 2013). The server predicts the probability of occurrence of regions such as α-helix, β-sheet and turns in secondary structure prediction. Eventually, NS mutation-driven relative alterations in structures and solvent accessibility were compared against the reference isolate.

#### 2.4.2. Protein dynamics analysis

Analysing protein structure-based alterations due to mutation determines the energy change value, significant to drive functional alterations. To achieve this, structure elucidation was done relying on the Modeller 9v8 (http://salilab.org/modeller/download_installation.html) (Sali and Blundell 1993), as no crystal structure of the studied (*P, L* and *G_NS_*) proteins were available. The FASTA sequences of proteins of Indian and Australian isolates were retrieved from the NCBI database and modelled. Further validation to predict various stereochemical properties was done by PROCHECK (Laskowski et al. 1993) and verify 3 D (Colovos and Yeates 1993). All models were visualized using Chimera tool (Pettersen et al. 2004).

For investigating the mutational consequences on the protein structural conformations, DynaMut server was employed (Rodrigues et al. 2018). The server is based on graph-based signatures in a consensus predictor algorithm and outperforms predicting the effect of mutations on protein flexibility and stability (p-value < 0.001). It works by uploading the modelled proteins to evaluate the impact of mutation in native protein structures based on dynamicity and protein stability resulting from vibrational entropy changes (ΔΔS_Vib_ENCoM) and differences in the free energy (ΔΔG). It uses a well-established normal mode analysis (NMA) to interpret and visualize the practicality of protein structural dynamics.

Additionally, analysis on altered conformations, evaluating their interatomic interaction due to investigated mutations was done. By utilizing the DynaMut server, results of the interacting molecules demonstrate how often mutation disrupts the interacting bonds, using a pairwise alignment algorithm (Bauer et al. 2019) by comparing against the wild type.

### 2.5. Virus isolation

Virus isolation has also been attempted in Vero and Baby hamster kidney (BHK-21), cell lines, respectively. The cell monolayer has been inoculated by the extracted PBMCs and serum of BEFV positive samples as previously performed (Chen et al. 2010; Zaher and Ahmed 2011). These were eventually passaged every eighth day and observed routinely for visible cytopathic effect (CPE). The supernatant was further investigated by RT-PCR amplification for virus presence.

## 3. Results

### 3.1. Clinical and haemato-biochemical evaluations

The outbreaks were episodically epizootic from July-October during 2019. The studied group comprised animals suspected of BEF based on the typical clinical signs and hemato-biochemical data. Most of the affected animals showed significant neutrophilia and lymphocytopenia in the hematological analyses. Similarly, blood biochemical analysis revealed decreased calcium concentrations and elevated creatinine kinase activities compared to normal, attributing to recumbency and lameness in these animals. Details of the hemato-biochemical analyses are depicted in Table S2. Although the infected animals almost exhibited full recovery within 3-10 days, they suffered significant milk losses as evidenced by lactation cessation in 22 out of 25 diseased animals extending up to 8-10 months.

### 3.2. Virus confirmation and sequencing

The PCR analysis showed expected-sized amplicons, confirming BEFV suspicion in 19 samples. Of these, a randomly picked sample was further utilized for complete genome sequencing. The PCR purified amplicons were sequenced and assembled, generating a near-complete genome of IND/IDR/BEFV/2019. Analogously, Illumina sequencing yielded similar outcomes with a *de novo* assembled 14,875 nt long contigs. Blastn search returned ∼95% identity to other published BEFV sequences. However, the sequence lacked a stretch of nt (15 at 5′ and 13 at 3′) as determined by consensus sequences of aligned genomes. Importantly, a perfect conservancy was displayed at 5’end, while 3′ revealed an average of 96%, with a maximum 99% to Israeli isolate (with the insertion of two nt in the missing region) and hence adopted to the Indian isolate (Identity matrix as shown in Table S3). Altogether, the first complete genome of IND/IDR/BEFV/2019 revealed a length of 14,903 nt with 33.37% GC content and deposited in GenBank under accession number MN905763.

### 3.3. Sequence analysis

**Figure 1.**
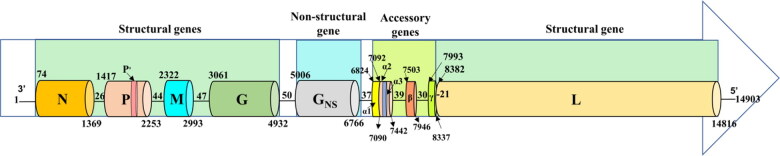
**Schematic diagram illustrating the genome organization of Indian BEFV isolate**. The complete length of single-stranded BEFV antigenome of 14,903 nucleotides (nt) along with the position of each terminal and gene, in the order from 3’ to 5’ polarity as 3’UTR (52 nt), nucleocapsid (*N*, 1328 nt), phosphoprotein (*P*, 858 nt), matrix (*M*, 691 nt), glycoprotein (*G*, 1897 nt), non-structural glycoprotein (*G_NS_,* 1785 nt), bicistronic alpha1(*α1*), alpha2 (*α2*) (630 nt), beta (*β*, 460 nt), gamma (*γ*, 400 nt) and large subunit of polymerase (*L*, 6470 nt) gene and 5’UTR (72 nt) are represented.

The aligned consensus sequences revealed a conserved genome pattern with each open reading frame (ORF) flanked by TI and TTP sequences and were separated by intergenic regions (Figure 1). A partial complementarity at 3′ leader (l) of 52 nt (1–52) and 5′ trailer (t) of 72 nt (14832–14903 nt) ends were observed, a characteristic property of all *Rhabdoviruses* (Dhillon et al. 2000). While few variations in the intergenic regions with nt insertions at *G_NS_*/*α1* and *β/γ* junctions in south-eastern isolates were observed, their significant difference in genome regulations requires experimental exploration. The details of each region are summarised in Table 1 and their sequences are depicted in Table S3.

**Table 1.** Represents the characterisation of complete Indian BEFV genome sequences depicting transcription initiation (TI) and transcription termination (TTP), coding sequences of genes with their respective positions.

Genes		mRNA transcription initiation	Coding sequence					mRNA transcription termination
***Gene***	***length (nt)***	***TI position***	***ORF length (nt)***	***Start sequence***	***Position***	***Stop sequence***	***Position***	***TTP position***
***N***	1328	53-58	1296	ATG	74	TAG	1367	1370-1380
***P***	858	1407-1412	837		1417	TAG	2251	2254-2264
***M***	691	2309-2314	672		2322	TGA	2991	2989-2999
***G***	1897	3047-3052	1872		3061	TAA	4930	4933-4943
***G_NS_***	1785	4994-4999	1761		5006	TAA	6764	6768-6778
***α***	638	6816-6821	267 (α1)		6824	TAA	7088	7443-7453
			351 (α2)		7092	TGA	7440	–
***β***	460	7493-7498	444		7503	TGA	7944	7942-7952
***γ***	400	7983-7988	345		7993	TAA	8335	8372-8382
***L***	6470	8362-8367	6435		8373	TAA	14814	14821-14831

The Indian sequence revealed the highest (∼96.53%) identity to isolates of the Middle East, followed by East Asia (∼91%) and lastly, Australia (89.84%). Further comparison of each terminus and the concatenated non-coding regions also followed a similar pattern. However, aa similarity ranged from 85.65% to 92.93%. All comparisons are represented in Table 2.

**Table 2.** Depicting the comparison of nucleotide (nt) and amino acid (aa) similarity between Indian BEFV isolate with all globally available BEFV complete genome sequences isolates, along with each gene, 3’UTR, 5’UTR with concatenated non coding region. Information on country from where it is isolated, accession number, complete sequence length is denoted.

Acc. no.	AF234533	KM276084	KY315724	MH756623	MH105245	MN078236	KY012742
**Country**	Australia	China	China	China	Thailand	Israel	Turkey
**Length (base)**	14900	14899	14941	14943	14943	14905	14901
**Genome nt (aa)**	89.85 (89.85)	91.18 (86.99)	91.48 (86.96)	90.91 (85.65)	90.97 (85.97)	96.56 (92.93)	95.24 (90.45)
N nt (aa)*	92.28 (98.38)	93.67 (99.54)	93.52 (99.30)	93.21 (99.54)	93.13 (99.54)	97.38 (99.54)	97.53 (100)
**P nt (aa)**	85.90 (80.94)	88.00 (87.41)	88.12 (87.41)	88.12 (87.41)	87.64 (86.69)	95.46 (94.96)	94.27 (91.73)
**M nt (aa)**	91.42 (95.96)	92.41 (99.10)	92.41 (98.65)	92.26 (99.10)	91.96 (99.10)	96.67 (99.10)	96.43 (99.10)
**G nt (aa)**	90.22 (94.86)	91.56 (96.95)	92.31 (97.27)	91.13 (96.63)	91.35 (96.79)	96.95 (98.88)	97.17 (99.20)
**G_NS_ nt (aa)**	89.37 (82.44)	90.68 (82.43)	91.14 (83.58)	91.03 (82.55)	91.25 (82.93)	94.88 (91.21)	96.53 (94.31)
**α1 nt (aa)**	88.39 (88.64)	91.02 (93.18)	91.02 (93.18)	89.51 (94.32)	89.51 (94.32)	89.57 (96.59)	96.20 (93.18)
**α2 nt (aa)**	87.18 (88.79)	88.89 (93.10)	89.74 (94.83)	89.74 (95.69)	90.03 (95.69)	93.73 (94.83)	96.87 (97.41)
**β nt (aa)**	88.74 (93.84)	91.44 (96.58)	91.89 (97.28)	93.24 (97.96)	93.24 (97.96)	93.24 (95.21)	97.07 (97.28)
**γ nt (aa)**	90.72 (99.12)	92.46 (97.37)	92.75 (97.37)	91.88 (97.37)	91.88 (97.37)	91.88 (95.61)	97.10 (99.12)
**L nt (aa)**	90.46 (96.22)	91.22 (96.50)	91.42 (97.34)	91.06 (97.25)	91.06 (97.25)	95.10 (97.81)	96.83 (99.11)
**3’UTR (nt)**	95.77	94.37	94.37	95.77	94.37	98.63	95.77
**5’UTR (nt)**	89.41	87.06	86.21	88.51	87.06	87.06	88.51
NCR (nt)**	85.71	86.99	86.96	85.65	85.71	92.93	90.45

Pairwise comparison of the complete genome of Indian BEFV isolate (Acc.no. MN905763) that was compared against all other complete sequences of BEFV is denoted. Light grey shade denotes for the coding regions whereas, darker shade for non-coding regions.

* The percent nucleotide identity is represented by the number without the bracket and the amino acid identity with the number within it in all genes except for non-coding region.

** It is the assembled concatenated non coding region of the BEFV complete sequence. Above numbers represents the values obtained after performing pairwise sequence alignment that calculated the distances of aligned sequences as done by Bioedit.

### 3.4. Phylogenetic analysis and genetic diversity

**Figure 2.**
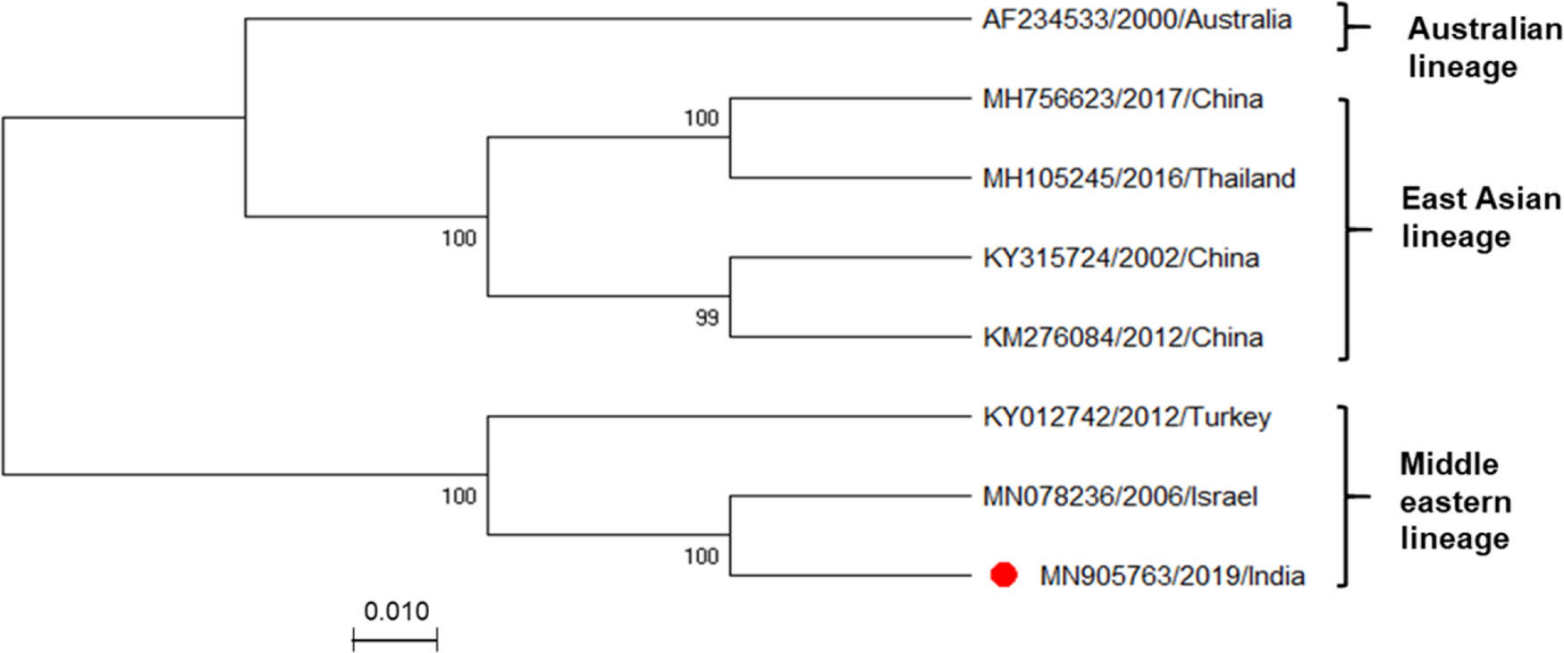
**Phylogenetic analysis of Indian BEFV isolate.** Phylogenetic analysis of complete Indian BEFV genome (marked with a red circle, n = 8) compared with corresponding globally available sequences. The maximum likelihood (ML) method and the Tamura-Nei model of nucleotide substitution were employed with bootstrap values (1,000 replicates) for tree construction. Bootstrap values are shown at the nodes representing the maximum likelihood bootstrap values and their subclades. There is a total of 14,959 positions in the final dataset. The GenBank accession numbers, along with the country name and year of the collection, are indicated for each virus.

Phylogenetic analysis clustered IND/IDR/BEFV/2019 with the Israeli and Turkish isolates of Middle Eastern lineage (Figure 2). The pairwise genetic distance of all corresponding sequences, estimated to be 0.03% to 0.10%, revealed high-level identity suggesting the constant evolutionary rate of BEFV. Additionally, the genetic distance had no clear correlation with geographical distance.

Next, we comprehensively analysed the genomic mutational accumulation specific to the Indian isolate, in context to unique nt and aa variations, revealing 76 substitutions (34 in non-coding and 42 in coding regions) and 30 substitutions (20 in structural proteins and 10 in non-structural proteins), respectively (Table 3). Except for *N, M, β*, and *γ* proteins with 100% identity, SN mutations were prominent in *P, G, G_NS_, α1, α2*, and *L* proteins, with few NS mutations observed in *P, L* and *G_NS_* proteins as well (Table 3). Interestingly, the third nt substitution of codon seemed most frequent for identified NS mutations. The results indicated the *L, P,* and *G_NS_* proteins to show the highest variation with 10, 9, and 8 mutations, respectively, that specifically included 4, 3, and 3 as NS mutations.

**Table 3.** Represents the number and type of unique variations/substitutions in the nucleotide and amino acid of all the genes with respect to their position. Additionally, Synonymous mutation (SN) and Non-Synonymous mutation (NS) that were observed within the Indian BEFV isolate in comparison to globally available sequences are also summarized.

Gene	Gene product	Unique nucleotide variation	Unique amino acid substitution	Substitution type with no.		Substituted amino acid position and change			
				SN	NS	SN		NS	
						Position	Change	Position	Change
**N**	Nucleoprotein	7	0	0	0	–	–	–	–
**P**	Phosphoprotein	14	9	5	4	59	P/T→ A	45	E/R → G
						154	V→I	115	S/L/F→
						178	I/V/P→L	152	P
						183	N/R→K	173	S→L
						272	I→V		K→N
**M**	Matrix protein	0	0	0	0	–	–	–	–
**G**	Glycoprotein	3	1	1	0	18	K → R	–	–
**G_NS_**	Non-structural Glycoprotein	25	8	5	3	16	T → N	490	E → G
						37	L→ M	514	E → K
						177	N→S	520	K → I
						380	I → V		
						504	L→V		
**α1**	Alpha 1 protein	4	2	2	–	51	A→ V	–	–
						83	K→ R		
**α 2**	Alpha 2 protein	4	1	1	0	40	E→ D	–	–
**β**	Beta protein	1	0	0	0	–	–	–	–
**γ**	Gamma protein	0	0	0	0	–	–	–	–
**L**	Large subunit of polymerase protein	18	10	6	4	12	H→N	1635	K→E
						1099	K→R	1937	K→N
						1564	E→D	2072	G/E→R
						1766	D→E		
						2044	L→ I		
						2058	K→R		
						2107	K→Q		

* SN depicts synonymous mutation and NS shows non-synonymous mutation. All amino acid is denoted by its standard symbol used universally.

### 3.5. Assessment of genomic NS mutations on protein dynamics

The NS mutations stand unique to the particular isolate that profoundly impacts the overall structural properties and biological function (Lewin et al. 2018). Our study utilized the *in-silico* approach to analyse their impact on various parameters affecting protein secondary and tertiary structures. Additionally, the overall impact due to these NS mutations were evaluated to seek their biological impact.

#### 3.5.1. Secondary structure and its mutational impacts

**Figure 3.**
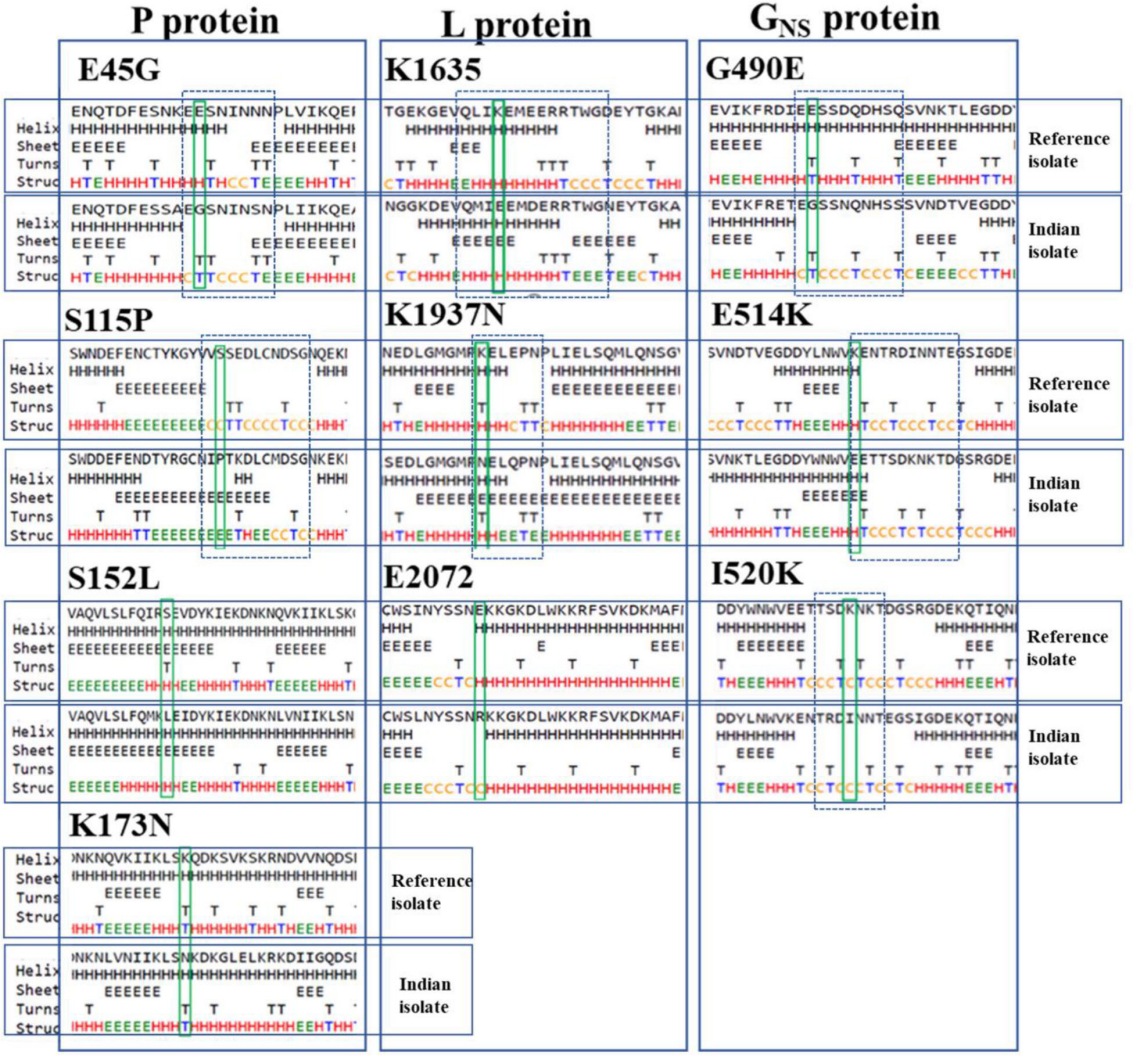
**Representation of the effects of non-synonymous (NS) mutations on protein secondary structure.** Depicts the prediction of the secondary structure due to change in mutation comparing Indian BEFV isolate to the Australian isolate (reference sequence) in *P, L* and *G_NS_* proteins. The rectangular box shows the mutant residue against the wild type. The position of the dashed box in the corresponding panels highlights the difference in secondary structure between Indian and Australian isolates.

The impact of these mutations on the secondary structure of mutant proteins was investigated. Our findings demarcated the changes observed in the mutant protein. It shows that except for two mutations, including S152L and K173N in *P* protein, all the remaining eight sites, E45G, S115P, in *P*; K1635E, K1937N, E2072R in *L* and G490E, E515K, K520I in *G_NS_* proteins induced changes in secondary structure. The detailed analysis revealed that mutants attained considerable alterations specifically at mutation sites (E45G, S115P in *P* and E2072R in *L*) or in the neighbouring residues (remaining all other mutants), indicating alterations in the local protein conformations in the secondary structure as compared to the reference isolate (Figure 3).

The significant alterations in *P* protein were depicted as few charged (E) and polar residues (S) were substituted by uncharged, non-polar, and hydrophobic residues (*G_NS_, P,* and *L*), though with little altered solvent accessibility. Likewise, in *L* protein, mutant replaced few positively charged residue with a negative or uncharged residue (K→E/N), with nonsignificant changes observed. In contrast, the substitution of negative to positively charged residue (E→R) may lead to the breakage of hydrogen bonds between the bases, thus altering their structures. All residues showed their solvent accessibility conversion from exposed to slightly buried (K→E/N) and converse (E→R). Similarly, the *G_NS_* protein displayed transitions only in the neighbouring residues resulting in altering the secondary structure. For solvent accessibility, a transformation from strongly exposed charged lysine to most buried hydrophobic isoleucine and negatively charged slightly exposed (K→I/E) residues was observed.

#### 3.5.2. Stability dynamics comparison of tertiary structure

**Figure 4.**
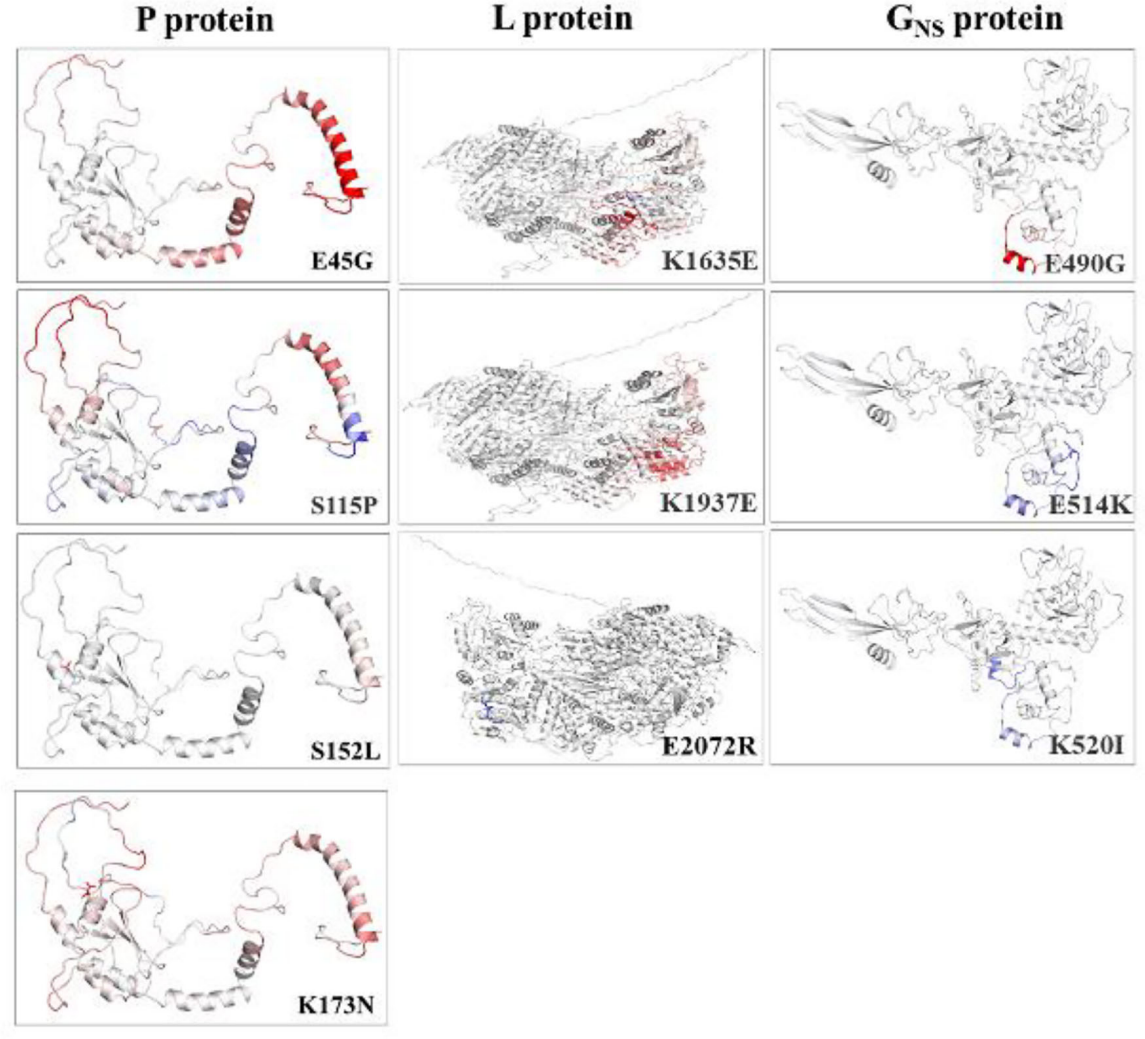
**Depicting the effects of non-synonymous (NS) mutations on protein structural dynamics.** Analysis of proteins stability and flexibility due to NS mutations. Visual representation of change in vibrational entropy energy between wild-type and mutant proteins. The colour change in amino acids is according to the ΔΔS_Vib_ENCoM value as a result of mutation. Red represents a gain in flexibility and blue indicates rigidification of the structure.

By analyzing the 3 D protein models in DynaMut server, we correlated the interpretation of secondary structure alterations to tertiary structure protein dynamics. All the built models were refined and validated, assuring their high quality for dynamics analysis. Our data inferred, most of the identified NS mutations resulted in destabilization with negative ΔΔG value and enhanced molecular flexibility with a positive ΔΔS_Vib_ENCoM value. Except for K520I and E514K mutants of G_NS_ protein with significantly higher positive ΔΔG (each as, −0.282 and −0.504 kcal/mol) and negative ΔΔS_Vib_ENCoM values (each as, 1.003 and 0.856 kcal.mol-1. K-1**)** demarcating enhanced stability and rigidity, respectively (Table 4) (Figure 4). The ΔΔS_Vib_ENCoM value represents an average of the protein configurational entropies within a single minimum energy landscape (Goethe et al. 2015). Whereas ΔΔG is the difference in ΔG of native and mutant type (Eriksson et al. 1992). Generally, ΔΔG positive value induces stabilization, and ΔΔS_Vib_ENCoM with negative value results in a decrease in flexibility. Additionally, other structure-based prediction tools such as mCSM, SDM, and DUET included as a module of the DynaMut server, further supported similar stabilization behavior, thus confirming our findings (Rodrigues et al. 2018).

**Table 4.** Representing the analysis based on the differences in ΔΔS_Vib_ENCoM and ΔΔG value due to mutation affecting the stability and flexibility of the mutated protein of Indian BEFFV isolate (MN905763) as compared to wild type reference sequence (AF234533).

S.no	Reference isolate	Indian isolate	Amino acid position	**ΔΔS_Vib_ ENCoM** (kcal.mol-1. K^-1^)	Effect (increasing)	**ΔΔG DynaMut** (kcal/mol)		Stability effect
**Phosphoprotein**								
1	E	G	45	0.954	Flexibility	−0.051	Destabilizing	
2	S	P	115	0.096	Flexibility	−0.682	Destabilizing	
3	S	L	152	0.046	Flexibility	−0.395	Destabilizing	
4	K	N	173	0.527	Flexibility	−0.393	Destabilizing	
**L protein**								
1	K	E	1635	0.398	Flexibility	−0.635	Destabilizing	
2	K	N	1937	0.082	Flexibility	−0.204	Destabilizing	
3	E	R	2072	−0.063	Flexibility	−0.052	Destabilizing	
**G_NS_ protein**								
1	E	G	490	0.487	Flexibility	−1.183	Destabilizing	
2	**K**	**I**	**520**	**-0.282**	**Flexibility**	**1.003**	**Stabilizing**	
3	**E**	**K**	**514**	**-0.504**	**Rigidity**	**0.856**	**Stabilizing**	

**Figure 5.**
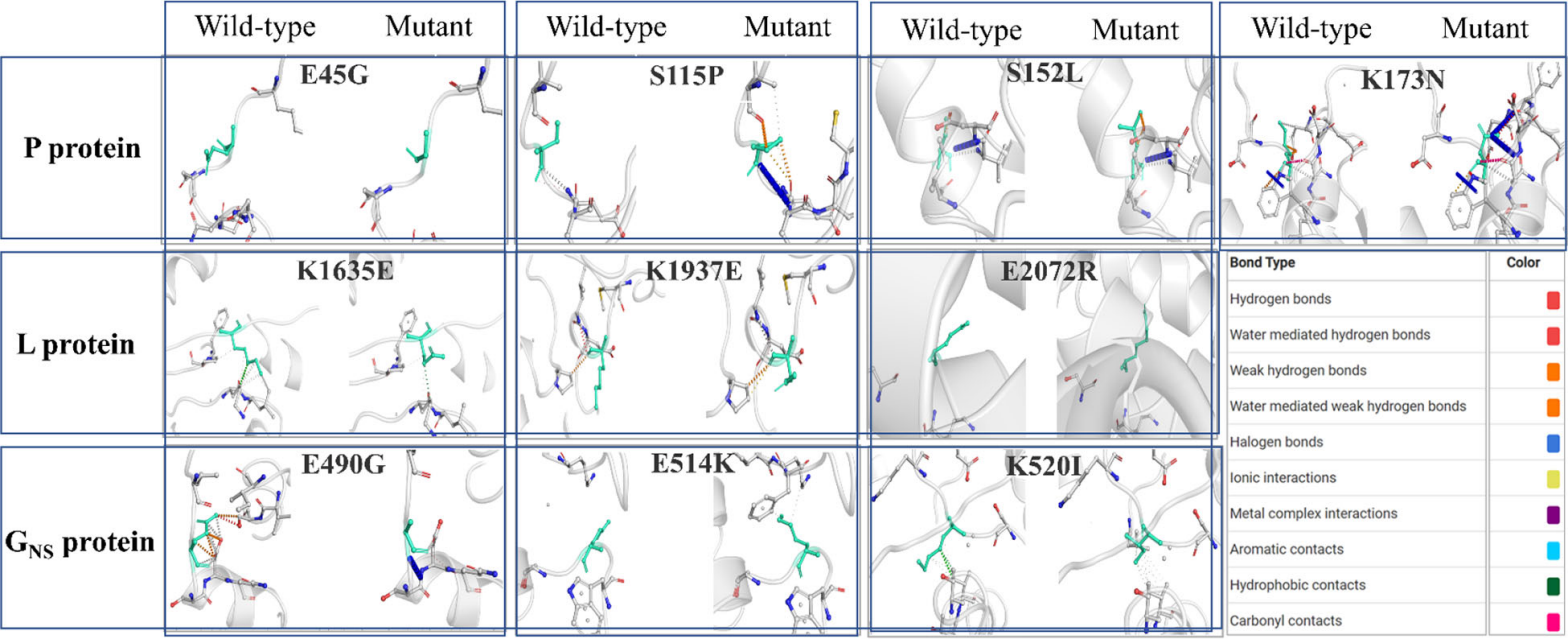
**Visualization of comparative prediction of interatomic interactions due to NS mutations.** The possible interatomic interaction rearrangements mediated by NS mutations in each of the proteins is represented. Wild-type and mutant residues are represented as light-green coloured sticks and all the surrounding residues involved in any type of interactions are denoted in different colours as mentioned in the box.

Furthermore, the significant relative variations in the mutants' stability and flexibility can investigate changes in protein confirmations. The comparison revealed significant alteration in the interatomic interaction among the variant and the wild-type. Major changes were observed in E490G and K514I mutants in *G_NS_* protein and S115P in *P* protein, while the rest showed slight alterations. The findings may contribute to explore the mutational influence on interatomic interactions of the proteins with their surrounding environment. All the mutated residues influenced the side chains altering the bond types, including H-bonds, halogen bonds, hydrophobic bonds, etc. That determines the interactions among residues existing in the surrounding area. The difference in structures representing variations is depicted in Figure 5.

Subsequently, utilising the PROVEAN tool, we predicted their corresponding biological impacts as determined by the pairwise sequence alignment scores. Two mutations in *P* protein (S152L, K173N) showed deleterious effect, while the rest had a neutral effect (Table 5).

**Table 5.** Represents the pairwise sequence alignment scores based on the comparative score of wild and mutated protein sequence and the predicted effect of such identified NS mutations on the biological function of the mutated proteins.

Variants in each protein	Score	Prediction
**P protein**		
E45G	−1.770	Neutral
S115P	2.619	Neutral
**S152L**	**-3.667**	**Deleterious**
**K173N**	**-3.381**	**Deleterious**
**L protein**		
K1635E	−0.230	Neutral
K1937N	−0.254	Neutral
E2072R	−0.500	Neutral
**G_NS_ protein**		
E490G	−1.657	Neutral
E514K	0.212	Neutral
K520I	1.090	Neutral

### 3.6. Virus isolation

The cell monolayer evidenced profound CPE from the fourth to fifth passage after 48 hours post-inoculation. However, PCR confirmation produced unclear results. Thus, an assumption of an unknown virus that co-exists and co-circulates predominates its adaptability in cell culture compared to BEFV. This inference requires future experimental research. Hence, virus isolation proved unsuccessful.

## 4. Discussion

The enhanced endemicity, combined with high morbidity and increasing case-fatality associated with BEFV infection, seriously attributes to enormous economic losses to the global dairy sector. As the world's leading dairy producer, India is at immense risk to BEFV. This necessitates a thorough understanding of BEFV isolates and genome to assess the pathogenicity. This study performed RT-PCR and NGS analysis of prevalent BEFV isolate, accompanied by a mutation-linked assessment of protein structures, crucial in understanding the viral pathogenesis, transmission, evolution, and antiviral therapy development.

The nearly complete genome obtained through molecular approach was completed by utilizing the terminal sequence conservancy pattern of global BEFV sequences, which determined the IND/IDR/BEFV/2019 isolate, the first Indian BEFV genome sequence. The 14,903 nt long full-genome was comparable to the Australian and Middle eastern isolates but recorded ∼ 40 nt shorter than East Asian isolates. All eight available complete genomes (including ours) maintain a conserved genome architecture pattern, length similarities, and deduced proteins suggesting essentiality for the propagation and dispersion of BEFV within the host populations.

This isolate has an expected conserved TI and TTP sequences pattern, vital to recruiting RNA-dependent RNA polymerase (RdRp) for independent transcription (Zeng et al. 1998). Moreover, the IGRs separating each gene are crucial determinants in transcription initiation and termination events (Das and Pattnaik 2004; Leyrat et al. 2011). The current isolate showed a certain variation in nucleotide length in IGRs at *G_NS_*/*α1* and *β/γ* junctions compared to the South-Eastern isolates, which might influence viral gene expression and replication, which needs further exploration.

Intriguingly, sequence and phylogenetic analysis revealed a close relatedness to Israeli and Turkish isolates, implying a common origin. The branching patterns coincide with the previous *P* and *G* genes based phylogenetic analysis, projecting its relative high pathogenicity (Yeruham et al. 2010; Abayli et al. 2017; Pyasi et al. 2020). Interestingly, no significant correlation between genotypes and geographical distance was observed. Despite the geographical closeness to Eastern and South-Eastern countries, the Indian isolate revealed the highest similarity to Middle Eastern isolates. As this region shares a significant portion of the livestock trade with India, it indicates a probable reason for the close relatedness of this cattle pathogen.

As geographical traversion of viruses may prompt certain adaptive responses in the form of mutations generating new variants that are more potent and fit (Pfeiffer and Kirkegaard 2005). Henceforth, we comprehensively scanned the IND/IDR/BEFV/2019 genome for unique mutations. We observed a prevalence of unique nt substitutions, with high codon degeneration, majorly contributing to silent or SN mutations (n = 20) and a few NS mutations (n = 10). The sequence conservation pattern followed *M > G > N > L > P* for structural proteins and *γ > β>α2 > α1 > G_NS_* for the non-structural proteins. Our results correlate well to other arboviruses (Domingo and Holland 1997; Pomeroy et al. 2008) that sustain constant viral evolution despite high substitution rates, thus similarly BEFV retained a single serotype characteristic worldwide (Dietzgen et al. 2017).

The highest 10 mutations were observed in *L*, followed by 9 in *P,* and 8 in *G_NS_* proteins that included few NS mutations. The *L* protein is the sole enzymatically active protein and is essential for genome transcription and replication. Similarly, *P* protein showed the highest divergence degree (relative to its size), supporting its significant role in genome evolution (Tordo et al. 1988; Leyrat et al. 2011). Since the rhabdovirus *N-P-L* complex regulates viral biological processes in collaboration (Das and Pattnaik 2004), experiencing mutations in any of these may presumably interfere with overall virus biology. Likewise, mutations in the *G_NS_* protein with understudied functions indicate an uncertain outcome.

We also examined unique NS mutations in the context of alteration to protein confirmations, contributing to the emergence of new genotypes. For instance, China, Taiwan and Turkey isolates (KY012742) demonstrated an elevated case fatality rate due to such mutations (Zheng and Qiu 2012; Abayli et al. 2017; Yanase et al. 2020). In total, 10 unique NS mutations comprising 4 in *P*, 3 in *L*, and 3 in *G_NS_* regions were structurally explored to infer any functional consequences (Cao et al. 2020)(Das and Pattnaik 2004). Studies suggest *L* protein to be highly conserved (Poch et al. 1989), while *P* protein shows the highest divergence degree (Leyrat et al. 2011). The computational analysis predicted two mutations in *P* (S152L and K173N) to be considerably deleterious and impact *L* protein's polymerase activities by interfering with *L-P*, protein-protein interaction functions (Cao et al. 2020) (Table 4). However, further *in vitro* analysis is needed to corroborate this observation. Similarly, mutations in the methyltransferase region of *L* could alter mRNA capping function.

Interestingly, our data on NS mutations in the corresponding protein revealed alteration to the structural stability. Most mutants' secondary structure analysis demonstrated the transition from charged to uncharged residues and hydrophilic to hydrophobic residue substitutions. Also, solvent accessibility decreased as substituted residues were buried deep in the protein structures compared to the earlier exposed ones. Eventually, we were able to link the aforesaid data to extrapolating the transition in the extremely vibrant protein tertiary structures by comparing it to the native type. The data revealed that most of the investigated NS mutations presumably resulted in the loss of rigidity and stability as is seen with positive ΔΔS_Vib_ENCoM and negative ΔΔG values, respectively. In contrast, *G_NS_* protein with E514K and K520I mutations are of concern as they stabilize conformations due to local helix backbone distortions (Cornish et al. 1994), leading to gain in rigidity. Further, most mutants showed significant alteration in the interatomic interaction compared to the wild-type.

However, our finding has limitations due to the unavailability of any crystal structure and limited knowledge of the functions of three proteins. Nonetheless, the Indian BEFV genome has acquired mutations that could potentially influence the protein structure. However, their influence on protein functions and viral virulency or transmission remains to be elucidated by performing wet-lab experiments.

## 5. Conclusion

The study presents the first complete genome sequence of Indian BEFV isolate and confirms its relatedness to Middle Eastern countries. Genome characterization revealed considerable genetic diversity with several unique mutations. Most of these mutations in *P, L* and *G_NS_* proteins were predicted to potentially impact the protein conformation and dynamics. Except for E514K and K520I mutants of *G_NS_* protein that achieved stabilized conformations and increased rigidity, all other mutants exhibited increased flexibility and destabilized effect on the structure. Mutations in *P* protein (S152L and K173N) are predicted to be deleterious for structural stability. As our study focused primarily on *in-silico* analysis, the inference from this could pave the way for prospective studies centered on experimental validation. Integrated knowledge of all these aspects with a larger sampling size would enhance diagnostics and vaccine development efforts of this understudied pathogen.

## Supplementary Material

Supplemental Material

## Data Availability

All the data supporting this research study's findings are openly accessible in the GenBank repository (https://www.ncbi.nlm.nih.gov/genbank) with Accession numbers: AF234533, KM276084, KY315724, MH756623, MH105245, MN078236, KY012742 and MN905763. The protein id’s of all the sequences investigated (*P, G_NS_* and *L* proteins): QOU09201, QOU09205, QOU09210, NP_065399, NP_065403, NP_065409.
