# Supplementary material for: Complete genome sequencing and assessment of mutation-associated protein dynamics of the first Indian bovine ephemeral fever virus (BEFV) isolate
Source: Vet Q. 2021 Oct 29;41(1):308–19. doi: 10.1080/01652176.2021.1995909 (PMC8567923; doi:10.1080/01652176.2021.1995909)
Supplement: Supplemental Material [file TVEQ_A_1995909_SM6370.zip › suppl_data/Table S1.docx]

**Table S1**

**Primer sequences used for RT-PCR amplification of complete BEFV genome sequence of Indian isolate**

| S.no | Primer sequence (5’→ 3’) | Positon (from/to) | Annealing temperature (°C) | Amplicon length (bp) |
| --- | --- | --- | --- | --- |
| 1 | TTCAACAGGTCTCTTTCCTTCA | 48-69 | 50 | 827 |
| 2 | TCATCTATTTCATTCCCCTCTTCC | 851-874 |  |  |
| 3 | CAGGACTCATGGCATTAGGTC | 742-762 | 50 | 1678 |
| 4 | GCTGTACCCCATACTAGCATAG | 2398-2419 |  |  |
| 5 | ATGCTCACCCTTTTTAAGAAAGGG | 2322-2345 | 52 | 1921 |
| 6 | AGGTCTGTATTCGCACCAAGCTC | 4226-4248 |  |  |
| 7 | ATGTTTAAGGTCCTAATAATTACTTTGCTAG | 3061-3091 | 51 | 2621 |
| 8 | TGCTAAGCTTAATCAACTCAAGTC | 5658-5681 |  |  |
| 9 | CCTCCTCCAAAGTGTGATTCC | 5399-5419 | 51 | 2300 |
| 10 | CATTTCTCATCACAAACTCTCCA | 7676-7698 |  |  |
| 11 | ATAATGCTAAAAGAGGAAGC | 7224-7243 | 52 | 1498 |
| 12 | ATATATGGTGGGAATCTTCCG | 8701-8721 |  |  |
| 13 | ATTACAATCTCAACTCCCC | 8521-8539 | 52 | 1504 |
| 14 | TCCCAATCTAAACCATAATC | 10005-10024 |  |  |
| 15 | ACGAGATACCTGATATGATTG | 9819-9840 | 52 | 1208 |
| 16 | TCTGATTGCTGGATTATG | 11010-11027 |  |  |
| 17 | AGGAACAATAAAGGGACTG | 10811-10829 | 53 | 1504 |
| 18 | TATAGAAGACATCTGACACC | 12295-12314 |  |  |
| 19 | AGATTGGGAAGATCAGATC | 12101-12119 | 53 | 1535 |
| 20 | CATGGAGTGGTCTATCATC | 13617-13635 |  |  |
| 21 | GAATTTGGTCAAAGGAATCAAAA | 13381-13403 | 50 | 1426 |
| 22 | TTTTGCAATTTCTTCCTCATACTG | 14783-14806 |  |  |

Depicting the primer sequences used for the amplification of the whole BEFV genome sequence with the detailed information of the primers sequence from 5’ to 3’direction, its’ position in the genome map, annealing temperature optimised for each pair (in degree) and amplicon length that will be obtained after PCR amplification.
