# Supplementary material for: Complete genome sequencing and assessment of mutation-associated protein dynamics of the first Indian bovine ephemeral fever virus (BEFV) isolate
Source: Vet Q. 2021 Oct 29;41(1):308–19. doi: 10.1080/01652176.2021.1995909 (PMC8567923; doi:10.1080/01652176.2021.1995909)
Supplement: Supplemental Material [file TVEQ_A_1995909_SM6370.zip › suppl_data/Table S3 corrected.docx]

**Table S3. Depicting Identity matrix of the partial 3’ terminal (60 bp) of Indian BEFV to all the globally available BEFV isolates**

| **Australia** | **China** | **Turkey** | **China** | **Thailand** | **China** | **Israel** | **India** |  |
| --- | --- | --- | --- | --- | --- | --- | --- | --- |
| **AF234533_Australia** |  |  |  |  |  |  |  |  |
| **KM276084_China** | 94.9 |  |  |  |  |  |  |  |
| **KY012742_Turkey** | 98.3 | 93.2 |  |  |  |  |  |  |
| **KY315724_China** | 94.9 | 100 | 93.2 |  |  |  |  |  |
| **MH105245_Thailand** | 96.6 | 98.3 | 94.9 | 98.3 |  |  |  |  |
| **MH756623_China** | 96.6 | 98.3 | 94.9 | 98.3 | 100 |  |  |  |
| **MN078236_Israel** | 96.6 | 94.9 | 94.9 | 94.9 | 96.6 | 96.6 |  |  |
| **MN905763_India** | 94.9 | 93.2 | 93.2 | 93.2 | 94.9 | 94.9 | 98.3 |  |

Mega -X platform was utilized with maximum likelihood method and the Tamura-Nei model for table construction. Values are based on the measure of genetic distance between the multiple aligned sequences being classified. Matrix attempts to construct an all-to-all matrix from the sequence query set describing the identity between each sequence pair.
