# Supplementary material for: Complete genome sequencing and assessment of mutation-associated protein dynamics of the first Indian bovine ephemeral fever virus (BEFV) isolate
Source: Vet Q. 2021 Oct 29;41(1):308–19. doi: 10.1080/01652176.2021.1995909 (PMC8567923; doi:10.1080/01652176.2021.1995909)
Supplement: Supplemental Material [file TVEQ_A_1995909_SM6370.zip › suppl_data/Table S4 corrected.docx]

**Table S4.** Representation of the site sequences of transcription initiation, transcription termination and intergenic sequences for all of the ten genes in BEFV sequences.

| **Genome region** | **Initiation signal** | **IGRs** | **Termination signal** |
| --- | --- | --- | --- |
| 3’leader/N | AACAGG | - | - |
| N/P | AACAGG | CCTTTAAATTCAATATAAGAAAACTC | CATGAAAAAAA |
| P/M | AACAGG | CCAGATAAGGAACTTGCAAATCAGGTCATCAATCAATTACAGTC | CATGAAAAAAA |
| M/G | AACAGG | TCATGCTAGATTAGGTAAGAATGAATAAAATTAAACAGAGTTAGGTT | CATGAAAAAAA |
| G/α1 | AACAGG | CTAATTTGCATCATGTGTTAACAAGGTAAACTTAAGACAAAATAAGAGGC | CATGAAAAAAA |
| α1/α2 | AACAGG | TAATAATAATAATCTGCACTGACCAATTAAAAATGGC | CATGAAAAAAA |
| α2/ β | AACAGG | CTAAGACATATAAGTAAGTTAGAAGTCTAGAAAAACAGC | CTTGAAAAAAA |
| β / γ | AACAGG | CTAGGTACATTATCAGGATCTACAAATGTT | CATGAAAAAAA |
| γ /L | AACAGG | ATGAATTTAAAATAATGATGAAAT | CATGAAAAAAA |
| L/5’trailer | - | - | - |
