# Supplementary material for: Complete genome sequencing and assessment of mutation-associated protein dynamics of the first Indian bovine ephemeral fever virus (BEFV) isolate
Source: Vet Q. 2021 Oct 29;41(1):308–19. doi: 10.1080/01652176.2021.1995909 (PMC8567923; doi:10.1080/01652176.2021.1995909)
Supplement: Supplemental Material [file TVEQ_A_1995909_SM6370.zip › suppl_data/TVEQ-2021-0008 table S2 final 2.docx]

**Table S2. Details of haemato-biochemical parameters of all the diseased animals as compared against the normal healthy cow.** All parameters revealed an altered feature in almost all of the animals, except the few that showed a value within the normal range.

| **S.no.** | **Haematological test** | | | | **Serum biochemistry** | |
| --- | --- | --- | --- | --- | --- | --- |
|  | ***Hb***  ***(g %)*** | ***Total leucocyte count ×10^9^/L*** | ***Differential leucocyte count*** | | ***Ionized calcium concentration***  ***mmol/L**** | ***Creatine kinase activity (U/L)**** |
|  |  |  | ***Neutrophiles*** | ***Lymphocytes*** |  |  |
| 1 | 8.2 | 10.8 | 64 | 62 | 0.394 | 1270 |
| 2 | 7.4 | 4.5 | 58 | 55 | 0.344 | 1202 |
| 3 | 7.2 | 6.5 | 54 | 43 | 0.311 | 932 |
| 4 | 11.2 | 8.4 | 46 | 66 | 0.361 | 1204 |
| 5 | 10.8 | 8.8 | 38 | 54 | 0.366 | 873 |
| 6 | 9.6 | 10.2 | 69 | 52 | 0.416 | 920 |
| 7 | 8.8 | 10.5 | 57 | 42 | 0.233 | 1200 |
| 8 | 8 | 6.5 | 48 | 65 | 0.327 | 980 |
| 9 | 9.3 | 7.7 | 65 | 62 | 0.250 | 790 |
| 10 | 8.4 | 7.3 | 64 | 64 | 0.194 | 890 |
| 11 | 11.8 | 9.7 | 70 | 63 | 0.233 | 1130 |
| 12 | 10.2 | 8.8 | 49 | 59 | 0.311 | 500 |
| 13 | 7.9 | 7.9 | 53 | 48 | 0.377 | 833 |
| 14 | 8.6 | 8.6 | 59 | 61 | 0.189 | 932 |
| 15 | 7 | 6.5 | 62 | 54 | 0.172 | 1204 |
| 16 | 10.8 | 7.7 | 66 | 62 | 0.200 | 873 |
| 17 | 8.8 | 10.8 | 56 | 52 | 0.377 | 920 |
| 18 | 11.2 | 4.5 | 52 | 40 | 0.272 | 270 |
| 19 | 11.4 | 4.3 | 38 | 64 | 0.161 | 1100 |
| 20 | 9 | 4.7 | 38 | 43 | 0.461 | 220 |
| 21 | 9.7 | 5.1 | 46 | 54 | 0.433 | 450 |
| 22 | 8.3 | 10.1 | 66 | 62 | 0.200 | 787 |
| 23 | 7.8 | 9.3 | 61 | 66 | 0.228 | 698 |
| 24 | 11 | 8.4 | 59 | 47 | 0.261 | 970 |
| 25 | 10.3 | 8.7 | 51 | 44 | 0.394 | 822 |
| Normal | 11.2 | 7.6 | 57 | 50 | 0.544 | 70 |

*Normal range of ionized calcium concentration: 0.538- 0.694 mmol/L; creatine kinase activity: 35-280 units/L
